# Metagenomic Quantification of Genes with Internal Standards

**DOI:** 10.1128/mBio.03173-20

**Published:** 2021-02-02

**Authors:** Emily Crossette, Jordan Gumm, Kathryn Langenfeld, Lutgarde Raskin, Melissa Duhaime, Krista Wigginton

**Affiliations:** a Department of Civil and Environmental Engineering, University of Michigan, Ann Arbor, Michigan, USA; b Ecology and Evolutionary Biology, University of Michigan, Ann Arbor, Michigan, USA; Pacific Northwest National Laboratory

**Keywords:** antimicrobial resistance, gene quantification, metagenomics

## Abstract

We demonstrate that an assembly-independent and spike-in facilitated metagenomic quantification approach can be used to screen and quantify over 2,000 genes simultaneously, while delivering absolute gene concentrations comparable to those for quantitative PCR (qPCR). DNA extracted from dairy manure slurry, digestate, and compost was spiked with genomic DNA from a marine bacterium and sequenced using the Illumina HiSeq4000. We compared gene copy concentrations, in gene copies per mass of sample, of five antimicrobial resistance genes (ARGs) generated with (i) our quantitative metagenomic approach, (ii) targeted qPCR, and (iii) a hybrid quantification approach involving metagenomics and qPCR-based 16S rRNA gene quantification. Although qPCR achieved lower quantification limits, the metagenomic method avoided biases caused by primer specificity inherent to qPCR-based methods and was able to detect orders of magnitude more genes than is possible with qPCR assays. We used the approach to simultaneously quantify ARGs in the Comprehensive Antimicrobial Resistance Database (CARD). We observed that the total abundance of tetracycline resistance genes was consistent across different stages of manure treatment on three farms, but different samples were dominated by different tetracycline resistance gene families.

## Abstract (precis)

qPCR and metagenomics are central molecular techniques that have offered insights into biological processes for decades, from monitoring spatial and temporal gene dynamics to tracking ARGs or pathogens. Still needed is a tool that can quantify thousands of relevant genes in a sample as gene copies per sample mass or volume.

## INTRODUCTION

A high-throughput, quantitative, gene-level screening tool is needed for studying dynamic, complex, and diverse microbial communities and the biological processes they perform. Quantitative PCR (qPCR) is widely used to measure the absolute concentrations of short segments of nucleic acid sequences, which serve as proxies of organisms or genes. This approach has been critical in a wide range of applications including assessing the relative roles of different microorganisms in nitrification and denitrification in wastewater treatment (1), the abundances of viruses in wastewater following outbreaks (2), and the impact of antibiotic use on antimicrobial resistance gene (ARG) abundances in livestock manure (3). However, qPCR is capable of targeting only limited sequences at a time, and primer bias, sensitivity, and specificity can confound results (4, 5). These aspects of qPCR limit our ability to compare, between samples and studies, the composition of microorganisms and genes in their community context.

Metagenomic sequencing has emerged as a powerful tool to study the structure and functional capacity of microbial communities. Metabolic gene databases, such as the carbohydrate-active enzymes database (CAZy), have facilitated gene classification from metagenomes for diverse applications ranging from evaluating the gut microbiome colonization in infants (6) to studying enrichment of cellulases in bioreactors for bioenergy production (7). Virulence gene databases, such as the Virulence Factor Database (VFDB), have enabled the development of metagenomic pathogen screening tools applied in a variety of settings from food safety to wastewater (8, 9). Although metagenomic analyses provide a comprehensive inventory of the genes and organisms that are present in samples, the data are compositional, and results are typically reported as relative abundances. As a result, studies that perform metagenomic sequencing alone cannot report absolute gene abundances, which are essential in many studies, particularly those evaluating changes in a pathogen marker gene or ARG concentrations through food, water, waste, and air treatment processes. Instead, metagenomic studies are limited to reporting relative changes in community diversity or the enrichment of certain genes between samples by normalizing to total sequence reads (10), 16S rRNA gene reads (11), or single copy gene reads (12). In some cases, hybrid approaches convert relative abundance data from metagenomic analyses to absolute abundances by relying on ancillary analyses such as the number of cells measured by flow cytometry (13) or the number of 16S rRNA genes measured by qPCR (14, 15). These additional analyses require method optimization and can introduce biases. A more direct option for obtaining the absolute abundance of genes from metagenomic data involves spiking nucleic acid internal standards into samples before extraction or sequencing (16).

Incorporating internal standard spike-ins, as commonly used in analytical chemistry, can establish a ratio of metagenomic read abundance to gene copy concentration. Internal standard protocols were first applied to sequencing methods in transcriptomic experiments (RNA-seq) to quantify gene expression, identify protocol-dependent biases, and compare method sensitivity and reproducibility (17). Since then, protocols have been developed for 16S rRNA gene-amplicon (18) metagenome (19), and metatranscriptome (16) sequencing. Previous quantitative metagenomic spike-in studies have performed metagenome assemblies and then mapped short metagenomic reads to the assembled contigs (20). Such assembly-dependent methods are time-intensive and can fail to assemble genomes that harbor ARGs, particularly those of viruses (21) or plasmids and within genomic islands (22, 23), thus increasing false-negative detection rates. Additionally, assemblies can introduce bias toward highly abundant organisms, which are more likely to be assembled correctly (24).

In this study, we applied an assembly-independent, spike-in metagenomic approach for quantifying gene concentrations in environmental samples. We first quantified the recovery of the spike-in genes across different concentrations, %G+C contents, and gene sizes. We then compared the spike-in quantitative metagenomic approach with traditional gene quantification by qPCR and with a hybrid, spike-independent metagenomic method. Finally, we applied the approach to quantify ARG concentrations in dairy farm samples and demonstrated the benefit of quantifying broader groups of genes than is possible with targeted qPCR methods. Ultimately, we envision this high-throughput, quantitative, gene-targeted method will improve exposure and risk assessment modeling, optimize treatment processes for water, waste, and air, enhance microbiome-driven resource recovery or bioenergy production, and quantify the roles of microbes in host health and global nutrient and carbon cycling.

## RESULTS

### Equation for assembly-independent, absolute gene quantification using spike-in normalization.

Genomic DNA of a marine bacterium, Marinobacter hydrocarbonoclasticus (ATCC 700491) was spiked into DNA extracted from environmental samples to determine the relationship between read counts and gene copy concentrations (see Fig. S1 in the supplemental material). We used genomic DNA from M. hydrocarbonoclasticus as our spike-in DNA because it is a marine microbe foreign to our samples. In our study, DNA was spiked after extraction to ensure that differences between spike-in and sample DNA recoveries were limited to sequencing and read mapping biases only, rather than to biases introduced during any prior sample processing steps. Prior to performing the DNA extraction and spike-ins on the samples compared in this study, we assessed extraction recoveries and bias across different manure matrices using a Gram-positive bacterium and a Gram-negative bacterium (see Text S1 and Fig. S2 in the supplemental material). Mean recoveries of spike-in Gram-positive and Gram-negative organisms ranged from 75 to 110% and did not differ significantly (*P* value = 0.27; Fig. S2).

10.1128/mBio.03173-20.1 FIG S1 Spike-in experimental and bioinformatic approach. Download FIG S1, DOCX file, 0.2 MB. Copyright © 2021 Crossette et al. 2021 Crossette et al. This content is distributed under the terms of the Creative Commons Attribution 4.0 International license.

10.1128/mBio.03173-20.2 FIG S2 Extraction recovery observed for raw manure and land-applied manure slurry. Download FIG S2, DOCX file, 0.1 MB. Copyright © 2021 Crossette et al. 2021 Crossette et al. This content is distributed under the terms of the Creative Commons Attribution 4.0 International license.

10.1128/mBio.03173-20.8 TEXT S1 Extraction recovery analysis. Details of the approach to evaluate extraction recovery. Download Text S1, DOCX file, 0.01 MB. Copyright © 2021 Crossette et al. 2021 Crossette et al. This content is distributed under the terms of the Creative Commons Attribution 4.0 International license.

The average ratio of the known spike-in gene copy concentration to gene length-normalized counts of mapped reads was calculated. This ratio was defined as the spike-in normalization factor, η:

(1) $$\text{η}=\frac{1}{n}\sum_{i}^{n}\frac{c_{\mathrm{s,i}}}{z_{\mathrm{s,i}}/L_{\mathrm{s,i}}}$$

where *n* is the total number of genes in the *M. hydrocarbonoclasticus* genome, *c_s_*_,_*_i_* is the known spike-in gene copy concentration for each gene *i* in the *M. hydrocarbonoclasticus* genome (gene copies/microliter DNA extract) and $\frac{z_{\mathrm{s,i}}}{L_{\mathrm{s,i}}}$ is the length-normalized read count (reads/base pair) for gene *i*. In this approach, we assume the relationships between gene copy concentrations and length-normalized read counts are consistent between the target genes and spike-in genes. We confirmed the gene recovery was robust across gene lengths and %G+C contents and different spike-in gene abundances by observing read mapping rates using different tools and settings (Text S2, Fig. S3, and Fig. S4).

10.1128/mBio.03173-20.3 FIG S3 (A) Variation in spike-in gene recoveries (*z_s_*_,_*_i_*/*z*_tot_*L_s_*_,_*_i_*)/*c_s_*_,_*_i_* using four mapping approaches. The units of the *y* axis are 1/gene copies/(μl)(base pairs) where the gene copies/μl are the known copies per DNA extract volume and base pairs are based on the length of the gene. (B and C) Spike-in gene recovery of *M. hydrocarbonoclasticus* genes from the metagenome across gene lengths (B) and %G+C contents (C). Genes are binned into 20 quantiles, and extended lines represent the interquartile range for each bin. Download FIG S3, DOCX file, 0.2 MB. Copyright © 2021 Crossette et al. 2021 Crossette et al. This content is distributed under the terms of the Creative Commons Attribution 4.0 International license.

10.1128/mBio.03173-20.4 FIG S4 (A) Spike-in gene recovery of internal standards across different concentrations of spike-in. (B) Correlation of relative number of reads mapped to spike-in genes (*z_i_*/*z*_tot_) to spike-in gene mass abundance (mass of spike-in DNA/total DNA) for the four same spike-ins in Fig. S4A. Dotted line, theoretical 1:1 relationship; solid line, linear regression. Analysis performed using the farm C digester sample. Download FIG S4, DOCX file, 0.1 MB. Copyright © 2021 Crossette et al. 2021 Crossette et al. This content is distributed under the terms of the Creative Commons Attribution 4.0 International license.

By multiplying η by the target gene’s length-normalized read counts ($\frac{z_{t}}{L_{t}}$, reads/base pair), we can predict the unknown concentration of our target gene ($\hat{c_{t}}$, gene copies/volume of DNA extract):

(2) $$\hat{c_{t}}=\eta {\left(\frac{z_{t}}{L_{t}}\right)}_{\;}$$

However, we ultimately aimed to determine the number of copies of the target gene per mass or volume of sample. For this, the target gene concentration was multiplied by the volume eluted during DNA extraction (*V*_eluted_, in microliters) to obtain the total copies of the target gene extracted, which was then divided by the mass (or volume) of the sample extracted:

(3) $$\frac{\text{copies, target}}{\text{sample mass}}=\hat{c_{t}}{\left(\frac{V_{\text{eluted}}}{\text{sample mass}}\right)}_{\;}$$

Here, it is assumed that spike-in genes are recovered at the same rate as the target genes in the sample. The spike-in facilitated approach establishes a relationship between read abundances and gene concentrations; we are therefore able to directly compare gene abundances between samples without corrections for average genome sizes or single copy gene concentrations.

Last, we found that the dynamic range of the relationship between gene concentration and read abundance spanned over 3 orders of magnitude and was consistent over different sequencing depths by spiking aliquots of a sample with different concentrations of the internal standard (Fig. S4). We found that the limit of detection corresponded to about 3 × 10^4^ gene copies/mg sample (Text S2).

10.1128/mBio.03173-20.9 TEXT S2 Read mapping validation. Download Text S2, DOCX file, 0.02 MB. Copyright © 2021 Crossette et al. 2021 Crossette et al. This content is distributed under the terms of the Creative Commons Attribution 4.0 International license.

### Agreement between sequencing- and spike-independent approaches validates our method.

**FIG 1:**
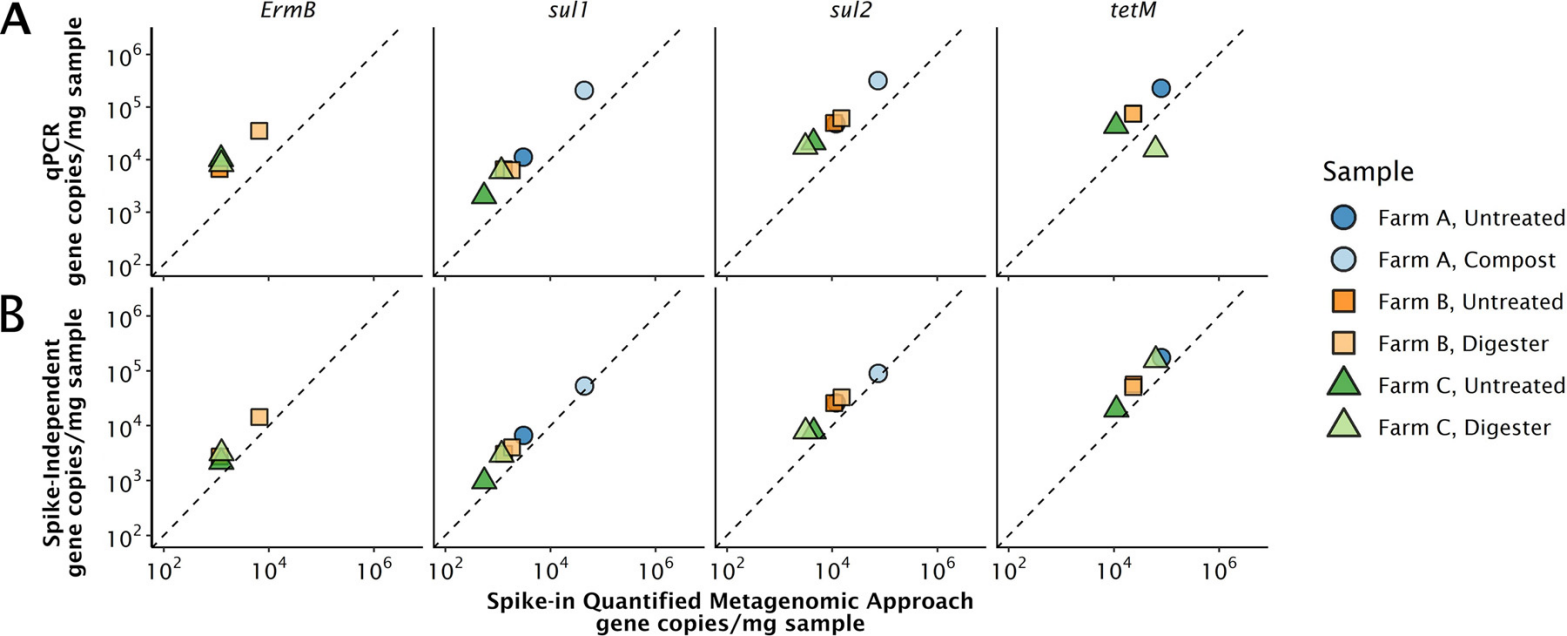
Comparisons of the gene quantification approaches using GROOT for assigning reads to resistance genes. The dotted line is a 1:1 line that represents theoretical perfect correlation between approaches. (A) Spike-in quantified metagenomic absolute abundance approach versus qPCR. (B) Spike-in quantified metagenomic absolute abundance approach versus spike-independent, 16S rRNA gene-based metagenomic approach. *ermB* was not detected in the farm A samples by the quantitative metagenomic approach, but it was detected in the farm A compost sample by qPCR. *tetG* was detected by qPCR in all samples, but not by the quantitative metagenome approach.

We compared gene quantities measured with the spike-in quantitative metagenomic approach to those measured with qPCR and a hybrid, spike-independent metagenomic quantification approach. We used six manure samples from different farms and treatment stages (untreated, composted, or digested). Five target genes, *tetM*, *tetG*, *sul1*, *sul2*, and *ermB*, were chosen because they have frequently been quantified in environmental samples and primer sets are available (25–29). In the quantitative metagenomic approach, reads were assigned to ARGs in the Comprehensive Antimicrobial Resistance Database (CARD) using graphing resistance out of metagenomes or “GROOT” (Fig. 1A) (30). Additionally, read abundances were assigned to resistance genes in the MEGARes database using AMR++ (Fig. S5) (31).

10.1128/mBio.03173-20.5 FIG S5 Comparisons of the gene quantification approaches using AMR++ tool for assigning reads to ARG reference sequences. The dotted line is a 1:1 line that represents theoretical perfect correlation between approaches. (A) Spike-in-quantified metagenomic absolute abundance approach versus qPCR; (B) spike-in-quantified metagenomic absolute abundance approach versus spike-independent, 16S rRNA gene-based metagenomic approach. *ermB* was not detected in the farm A, Samples with AMR++ but was detected in the farm A compost sample with qPCR. *tetG* was detected by qPCR in all samples but not by the quantitative metagenome approach. Download FIG S5, DOCX file, 0.2 MB. Copyright © 2021 Crossette et al. 2021 Crossette et al. This content is distributed under the terms of the Creative Commons Attribution 4.0 International license.

In the hybrid, spike-independent metagenomic quantification approach, 16S rRNA gene concentrations are measured in each sample using qPCR. Then, target read counts are divided by 16S rRNA gene read counts (14, 15). This approach assumes that the target gene/16S rRNA gene quotient is equivalent for metagenomic sequencing and qPCR and is computed as follows:

$$\frac{\text{ target gene copies}}{\text{sample mass}}=\left(\frac{z_{\mathrm{s,i}}}{z_{\text{16S rRNA}}}\right)\left(\frac{\text{16S rRNA gene copies}}{\text{sample mass}}\right)$$

where *z_s,i_* is the number of reads mapping to the target gene, *z*_16S rRNA_ is the number of reads mapping to a 16S rRNA sequence, and the 16S rRNA gene copies/sample mass ratio is determined by qPCR.

*tetG* was not detected using the metagenomic approach in any of the samples but was detected with qPCR with abundances ranging from 1,000 to 2,400 copies/mg of sample, corresponding to about 2,000 to 6,000 copies/μl extract. *ermB* was not detected by the metagenomic approach in the farm A samples, but was detected in the farm A untreated sample with qPCR at 900 copies/mg sample. In these samples, we are approaching the method detection limit of the quantitative metagenomic approach (∼3 × 10^4^ gene copies/mg sample), but not that of qPCR (2 to 8 copies/mg sample, see Text S2 and Table S1 in the supplemental material).

10.1128/mBio.03173-20.6 TABLE S1 qPCR primers used in this study with annealing temperatures, limits of detection (LOD), limits of quantification (LOQ), efficiencies, and *R*^2^ values. Download Table S1, DOCX file, 0.02 MB. Copyright © 2021 Crossette et al. 2021 Crossette et al. This content is distributed under the terms of the Creative Commons Attribution 4.0 International license.

On average, qPCR quantities were 22% greater than those using the quantitative metagenomic approach, with *tetM* as a visible outlier. Specifically, the spike-in quantitative metagenomic approach predicted a fourfold-greater concentration of *tetM* than qPCR in the farm C digester. This discrepancy between approaches for *tetM* could result in major differences in study conclusions. For example, if the qPCR assay was used to measure how *tetM* concentrations changed between the farm C untreated and digester manures, one would observe a 95% decrease in *tetM* concentration. Using the spike-in quantitative metagenomic approach, however, one would observe a 140% higher concentration of *tetM* in the digester sample than the untreated sample (Fig. 1A). These patterns were also observed with AMR++ using the MEGARes database (Fig. S5).

GROOT and AMR++ tools use different approaches to reduce ambiguous mapping to resistance genes. Specifically, AMR++ employs the “ResistomeAnalyzer” algorithm which removes sparse alignments at thresholds that can be set by the user. GROOT uses variation graph representation of a user-specified database that stores shared gene sequences as graphical nodes, reducing ambiguous mapping to homologous gene regions. We therefore hypothesized that the incongruence observed for the *tetM* gene was due to qPCR primers failing to capture the diverse *tetM* genes in the digester manure sample. To test this hypothesis, we evaluated the read mapping patterns to *tetM* and *sul1* reference genes using Bowtie2 (32) and the Integrative Genome Viewer software (33). We included *sul1* in the analysis because it is a highly conserved gene sequence; therefore, we expected mapped reads to perfectly match the reference gene sequence. Six single nucleotide variants (SNVs) were observed in the reads mapping to the 19-bp *tetM* forward primer sequence. When the allele frequencies at the primer binding sites were quantified (Table S2), 99% of *tetM* mapped reads from the farm C digester had a mismatch at five of the six SNVs in the primer sequence. In the other samples, between 60 and 80% of the mapped reads had a mismatch at the same primer SNVs (Table S2). Incongruencies in primer binding sites and metagenomic reads were not observed for the *sul1* primer set. This analysis demonstrates that the *tetM* primers likely did not capture the diversity of this gene. As a result, the *tetM* qPCR assay underestimated *tetM* concentrations, especially in the farm C digester sample. In other words, the spike-in quantitative metagenomic method resulted in more reliable absolute *tetM* abundances than qPCR because it did not rely on primer design and *a priori* knowledge of sequence diversity.

10.1128/mBio.03173-20.7 TABLE S2 Allele frequencies of single nucleotide variants (SNV) compared to the *tetM* forward primer sequence aligned using Bowtie2. The base in the forward primer and position are provided as the column headers. Depth refers to the average read abundance. Download Table S2, DOCX file, 0.01 MB. Copyright © 2021 Crossette et al. 2021 Crossette et al. This content is distributed under the terms of the Creative Commons Attribution 4.0 International license.

To further assess the reproducibility of the spike-in quantitative metagenomic method, we compared the estimated absolute concentrations to a hybrid, spike-independent, 16S rRNA gene-based quantitative metagenomic approach (Fig. 1B). The spike-independent approach has been used previously for absolute quantification of ARGs in a river system (14) and markers of opportunistic pathogens in a drinking water distribution system (15). Percent differences between the hybrid, rRNA gene-based approach and the spike-dependent quantitative approach are a function of the two normalization factors, since the number of reads mapping to each target is the same for each approach. The limits of detection are the same for these approaches since they are both determined by read counts. Differences ranged from 13% to 21% between the spike-independent and spike-dependent approach, except for the farm A compost sample, which had a 4% difference (Fig. 1B).

Cross-validating our approach to sequencing-independent qPCR assays and a hybrid, spike-independent metagenomic approach for the five ARG targets validated that our method generates values comparable to established gene quantification tools. Although the spike-in metagenomic approach had higher detection limits than qPCR, it overcomes biases caused by primer specificity.

### Spike-in metagenomic approach facilitates quantitative screening of diverse gene families.

In six dairy farm manure samples, the spike-in metagenomic approach enabled the quantification of all genes in CARD. This is not feasible using traditional qPCR since each gene would require a validated set of primers and standard curves. Out of the 2,617 genes in CARD, 411 genes were detected in the six dairy manure samples using GROOT. The total number of different ARGs detected in each sample ranged from 62 to 361.

**FIG 2:**
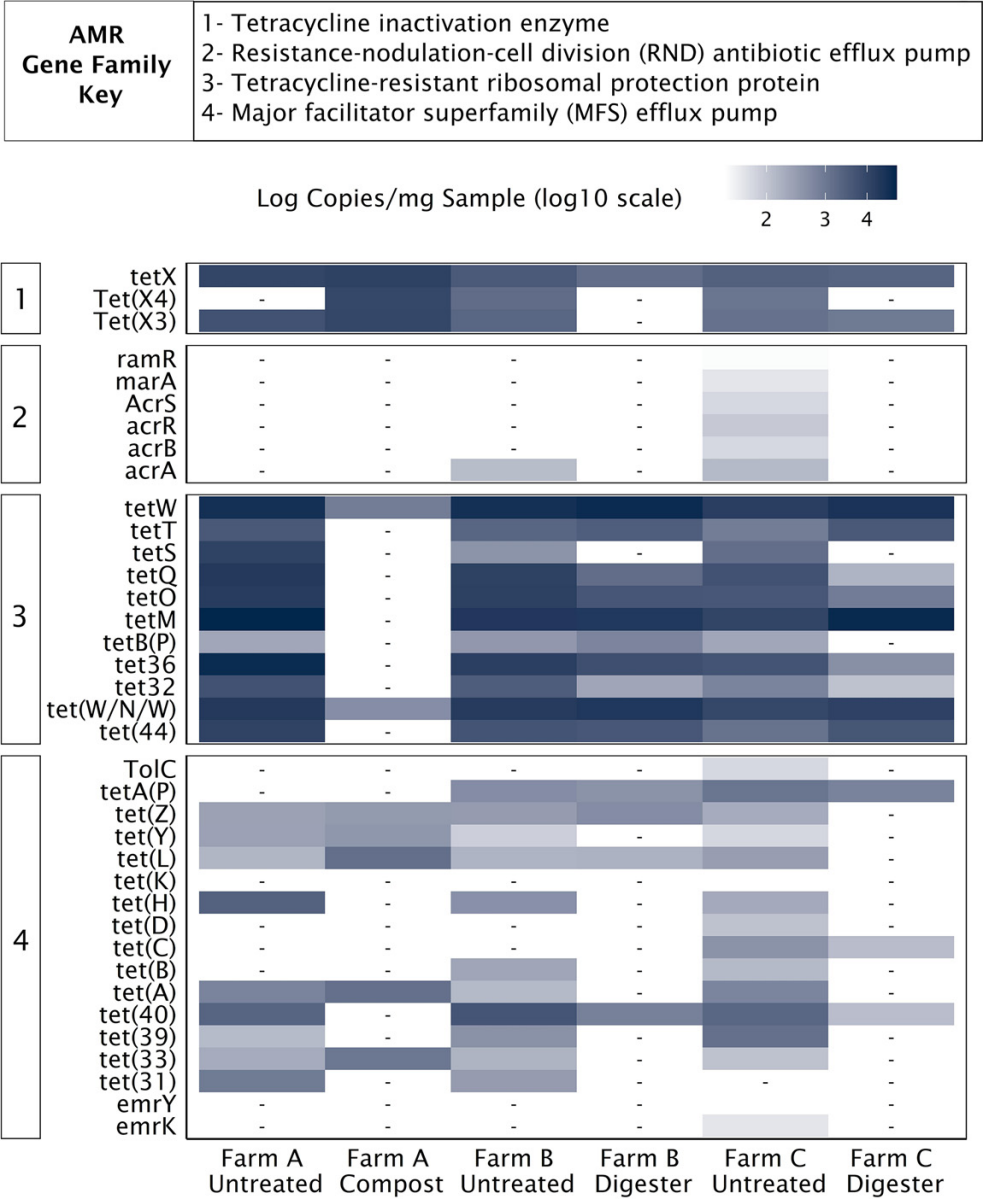
Absolute concentrations of all genes that confer resistance to tetracycline in six different dairy farm samples. -, not detected (no reads mapping to target).

To illustrate the diversity of genes that can be detected and quantified with a single approach, we leveraged the “confers_resistance_to_antibiotic” relation in the CARD ontology to extract genes within the tetracycline drug class. We then assessed the diversity and absolute abundance of these tetracycline resistance genes across broad gene families in each sample (Fig. 2). Of the 93 tetracycline resistance genes matching the “confers_resistance_to_antibiotic” ontology, 37 were detected in our samples with genes detected from four of the seven resistance gene families (Fig. 2). Genes were not detected from the small multidrug resistance antibiotic efflux pump, ATP-binding cassette ribosomal protection protein, and ATP-binding cassette efflux pump families in the manure samples. Interestingly, the sums of all the tetracycline resistance gene concentrations in each sample were all within 1 order of magnitude, ranging from 3.8 × 10^4^ to 3 × 10^5^ copies per mg sample (Table 1). However, different resistance gene families dominated the tetracycline resistance gene concentrations within different sample groups. Tetracycline resistance ribosomal protection proteins were the most abundant gene family, comprising 82 to 97% of the total tetracycline resistance genes in all samples except the compost sample from farm A. In this sample, tetracycline inactivation enzymes dominated the resistance profile, comprising 85% of the total tetracycline resistance gene concentrations (Fig. 2; Table 1). These data demonstrate that no single ARG could have been selected to represent the total tetracycline resistance abundances. For example, targeting just one or two tetracycline resistance genes with qPCR would have inadequately assessed the impact of residual concentrations of tetracyclines or how effective a manure treatment strategy had been at reducing the quantity of resistance genes within a drug class.

**TABLE 1:** Total abundance of tetracycline resistance genes, organized by CARD gene family, in six different dairy farm samples^*a*^

AMR gene family	Total abundance of tetracycline resistance genes (copies/mg sample)					
	Farm A, untreated	Farm A, compost	Farm B, untreated	Farm B, digester	Farm C, untreated	Farm C, digester
Major facilitator superfamily (MFS) antibiotic efflux pump	7.3 × 10^3^	4.5 × 10^3^	7.3 × 10^3^	1.7 × 10^3^	6.8 × 10^3^	9.1 × 10^2^
Resistance-nodulation-cell division (RND) antibiotic efflux pump			1.2 × 10^2^		4.4 × 10^2^	
Tetracycline-resistant ribosomal protection protein	2.7 × 10^5^	1.3 × 10^5^	1.4 × 10^5^	1.2 × 10^5^	5.6 × 10^4^	1.2 × 10^5^
Tetracycline inactivation enzyme	1.6 × 10^4^	3.2 × 10^4^	7.3 × 10^3^	1.5 × 10^3^	5.1 × 10^3^	3.2 × 10^3^

**Total gene abundance**	**3.0 × 10^5^**	**3.8 × 10^4^**	**1.5 × 10^5^**	**1.3 × 10^5^**	**6.8 × 10^4^**	**1.2 × 10^5^**

a The total gene abundance is shown in boldface type.

### Changes in spike-in-based absolute abundances and relative abundances through treatment.

Our samples comprised untreated and treated manure samples from three dairy farms; two treated samples were collected from anaerobic digesters, and one treated sample consisted of compost. Thus, this study provided an opportunity to evaluate the degree to which between-sample relationships in gene levels compared between the relative and absolute quantities.

**FIG 3:**
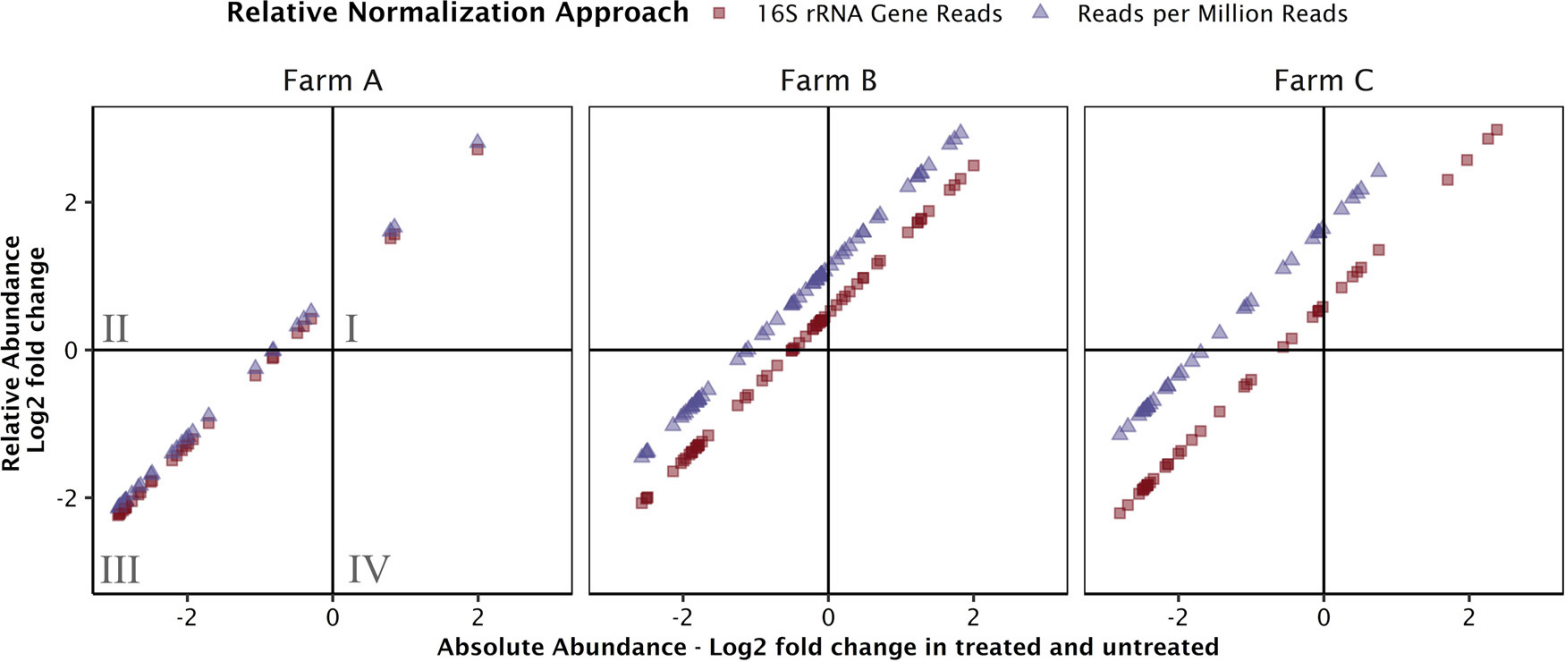
Comparing relative versus absolute abundances of resistance genes between treated and untreated manure samples detected with GROOT. Each point represents the log_2_ fold change in abundance of a gene between untreated and treated samples. Red squares compare fold changes determined by relative abundances normalized by 16S rRNA gene reads to our fold changes in absolute abundance using the spike-in quantitative metagenomic approach. Blue triangles compare fold changes in relative abundance normalized by library size to fold changes in absolute abundances. If a point falls in quadrant II (quadrants labeled in farm A plot), a positive log_2_ fold change was observed with relative abundance and a negative log_2_ fold change was observed with absolute abundance.

The number of metagenomic reads mapping to a target gene is used to determine both relative and absolute abundances of that gene in the sample. However, the normalization parameter is different between approaches. The simplest normalization parameter is the library size, or total number of reads generated in a sequencing run, though the approach poorly resolves log fold changes between samples (19). Another normalization parameter is the total number of reads mapping to 16S rRNA or single copy genes (34). These relative abundance approaches approximate the abundance of reads relative to bacterial and archaeal biomass. In contrast, the normalization factor in the absolute abundance spike-in approach derives from the relationship between gene concentration and read abundance established by a spike-in standard. The normalization parameter is the product of all terms in equation 3, save the number of reads mapping to the target. Between-sample comparisons in ARG abundances quantified by both relative and absolute normalization demonstrated that different approaches can predict conflicting directionality of change for genes for which the change in abundance is small (Fig. 3). In the sample pairs from farms A, B, and C, there were 42, 71, and 49 total genes detected in both samples, respectively. Between the untreated and treated sample pairs on farms A, B, and C, conflict in the direction of the change in abundance was observed in 3, 17, and 7 of those ARGs; specifically, decreases in absolute abundance were observed, whereas increases in 16S rRNA gene-normalized relative abundances were observed. The 1:1 correlation between observed changes in absolute abundance and relative abundance demonstrates that both approaches are functions of the reads recruited per target sequence. The intercept depends on the normalization parameter values for each sample. Plotting log fold changes in absolute gene abundances versus the library size-normalized relative abundances (Fig. 3, blue triangles) resulted in a *y* intercept greater than that of the absolute abundances versus the rRNA gene read normalized abundances (Fig. 3, red squares). A greater intercept means more ARGs fell within the quadrant II for the library size normalization approach; therefore, more ARGs were observed to increase in relative abundances while decreasing in absolute abundance. For those examples where the direction of the log_2_ fold change disagreed between relative and absolute abundances approaches, all changes were less than fourfold. When observed between-sample differences in abundance are small (log_2_ fold change of <2), distinguishing between abundance increases and decreases becomes noisier and more challenging to resolve.

## DISCUSSION

We demonstrated that assembly-independent, gene-targeted metagenome quantification with a genomic spike-in internal standard resulted in absolute gene quantities for thousands of genes simultaneously and at levels comparable to those obtained with qPCR and a spike-independent hybrid metagenomic approach. The spike-in approach circumvents the low-throughput and primer design challenges of qPCR and the bias-prone ancillary molecular methods required for hybrid approaches. Our approach requires only a spike-in internal DNA standard and a relevant gene database, such as CARD, CAZy, or a custom gene set of interest.

Previous quantitative sequencing studies have spiked DNA into samples prior to extraction (18, 20), into samples after cell lysis (35), and into nucleic acid extracts (19). Cellular internal standard spike-ins enable estimation of recovery from cell lysis to sequencing. However, each organism has a unique cellular morphology and susceptibility to lysis, so one or a handful of cellular spike-ins may not reflect extraction efficiencies for the diverse organisms in a sample (36). We spiked in *M. hydrocarbonoclasticus* genomic DNA into the DNA extracted from environmental samples prior to sequencing to directly observe spike-in recovery in the absence of these complex biases and compared extraction efficiently in separate experiments (see Text S1 in the supplemental material). We note that the same nucleic acid extraction efficiency issues are present with any gene quantification method.

The number of documented and catalogued genes will continue to grow as new samples are sequenced, as isolation and culturing of environmental strains reveal more diversity, and as organisms evolve. qPCR primers are designed for gene targets using the gene sequences available in a database at a specific point in time. The primers are often applied in future studies. Consequently, without constant primer redesign and evaluation, qPCR quantification methods lag behind novel gene diversity discovery. Spike-in metagenomic quantification, on the other hand, can provide absolute quantification comparable to qPCR while using the most up-to-date gene databases. Furthermore, archived metagenomes can be reanalyzed to quantify newly discovered genes as databases expand.

The number of genes that can be measured simultaneously in a sample using spike-in metagenomic quantification is limited by database completeness. In our study, we were able to simultaneously screen the 2,617 genes in CARD. qPCR assays, on the other hand, are limited by the number of targets they can include. ARG studies that employ qPCR, for example, commonly target between 5 and 20 genes per sample (26, 37–39). Although qPCR arrays have increased the throughput of qPCR and provided valuable insights on ARG profiles (28, 40), qPCR arrays without standard curves do not deliver absolute concentrations, are subject to the same primer specificity limitations as traditional qPCR, and are similarly limited by available knowledge on ARGs at the time of qPCR array design.

Determining detection limits and establishing detection ranges are critical for all quantitative methods. Here, spiking at different internal standard concentrations revealed that the linear range of detection spanned at least 3 orders of magnitude (Text S2). Spiking a standard corresponding to 0.1% of the total DNA in our sample revealed that we were reaching limits of detection for several genes at the order of 10^4^ gene copies/mg sample. The single genome spike-in used in this study meant that nearly all internal standard genes were present at the same gene copy concentration in a single sample. Staggered spike-in standards, which contain different sequences over a range of concentrations within one spike-in, can better characterize the quantification range in individual samples (18, 19, 35). Limits of detection, which are trumped by qPCR assays, are a primary limitation of this quantitative approach.

Our spike-in genome presented nonspecific mapping in regions of the *M. hydrocarbonoclasticus* genome that were homologous to genes of interest in the sample. For example, a gene in the *M. hydrocarbonoclasticus* genome shared 75% homology with an efflux pump-encoding gene in CARD. Interestingly, *M. hydrocarbonoclasticus* also shared >70% homology with three genes in the bovine genome. Synthetic DNA internal standards, as opposed to genomic standards, can eliminate nonspecific mapping (19). Similarly, assigning metagenomic reads to target genes of interest within databases can also result in false-positive and false-negative assignments. We emphasize that our approach is intended for high-throughput screening and is not appropriate for exploring and quantifying potential novel resistance genotypes that are not yet represented in databases. Other tools that leverage machine learning and functional metagenomics (41) or build models for specific genes (42) would be more appropriate in these applications.

This assembly-independent, spike-in-facilitated gene quantification is a fast, effective, and nontargeted approach to quantify known genes from microbial communities. This approach will be valuable when qPCR throughput and primer design limit the conclusions that can be drawn and when quantifying genes at low abundances is not required. The approach is especially useful in ARG research, where absolute quantification of diverse genes is imperative for evaluating technologies to reduce ARG abundances and informing models focused on antimicrobial resistance risk assessment.

## MATERIALS AND METHODS

### Sample collection.

One-hour composite samples were collected in June 2016 from dairy manure at three farms in New York following a protocol described previously (28). Samples included an untreated manure sample from blend pits at each farm and a posttreatment sample, either anaerobic digester effluent or compost. Samples were aliquoted into 15-ml centrifuge tubes, frozen at −80°C, and shipped overnight on dry ice to the University of Michigan.

### DNA extraction, internal standard spike-in, and sequencing.

DNA was extracted from approximately 250 mg (wet weight) of each sample in duplicate reactions using the QiaAMP PowerFecal kit (Qiagen, Germantown, MD). Extracted DNA was eluted in 100 μl of elution buffer following the kit’s protocol. Duplicate extractions were pooled. DNA concentrations were measured with a Qubit 2.0 fluorometer. The pooled DNA extracts were spiked with genomic DNA from *M. hydrocarbonoclasticus* (ATCC strain 700491D5, GenBank accession no. CP000514), obtained from ATCC (Manassas, VA) at 1% total DNA by mass. *M. hydrocarbonoclasticus* was resuspended following ATCC recommendations in molecular grade water, and the concentration was confirmed using the Nanodrop1000 instrument. This marine bacterium was selected due to its typical bacterial genome size (4,326,849 bp) and GC content (57%) and because it was unlikely to be present in manure samples. All six pooled and spiked DNA samples were sequenced with paired-end, Illumina (HiSeq4000) technology at the University of Michigan DNA Sequencing Core using PCR-free library preparation with a read length of 250 bp and an insert size of 450 bp. Total post-quality control (QC) reads per sample ranged from 5.1  × 10^8^ to 6.3 × 10^8^. To establish the linear quantification range of genes, replicates of one of the samples were spiked with different ratios of internal standard DNA to total community DNA (0.1%, 1%, and 10%, by mass).

### Bioinformatic approaches.

Reads were trimmed and checked for quality with BBDuk from the BBTools Package (43). The Comprehensive Antibiotic Resistance Gene Database (CARD) (44), the *M. hydrocarbonoclasticus* gene multifasta files from NCBI, 16S rRNA gene-specific small subunit (SSU) SILVA database (45), and the bovine genome (46) were downloaded on 20 December 2019, 24 October 24 2018, 24 May 2019, and 2 November 2018, respectively. Read mapping approaches were first evaluated by comparing observed to expected recoveries of *M. hydrocarbonoclasticus* genes. Specifically, Bowtie2 (32) (version 2.3.4.3) was run with default parameters, and kallisto (46) (version 0.46.0) was run with the “--single overhang” setting which counts reads only partially mapping to the end of reads and the bias correction setting “--bias” that can reduce the bias from larger reference genes where the effects of overhanging reads are less impactful. These tools were selected because they represent two common algorithms for fast short-read mapping, pseudoalignment, and Borrows-Wheeler transform-based read assignments. Read assignments were performed with both paired reads and unpaired reads. Average recovery across read lengths was assessed by clustering genes into 20 bins using quantile binning with the *Hmisc* package in R. One-way analysis of variance (ANOVA) was performed in R to compare distribution of average spike-in gene recoveries. After validating read mapping performance with the *M. hydrocarbonoclasticus* genes, kallisto in an unpaired mode with the “--single overhang” and “--bias” setting was selected to map reads to CARD and 16S rRNA gene-specific small subunit (SSU) SILVA database. Reads were mapped to the bovine genome with bbmap from the BBTools Package in the paired setting to assess host contamination. IGV software (version 2.7.2) was used to visualize read pile-ups to assess nucleotide variants at primer binding sites.

Two additional tools were used to assign reads to ARGs: (i) AMR gene-specific read assignment tools for antimicrobial resistance genes, AMR++ (31) and GROOT (30). Both tools apply unique approaches to reduce nonspecific mapping of reads to ARGs. GROOT was run with default parameters using CARD2020 as a reference except that the 50% reporting threshold was used. AMR++ (version 2.0.0) was performed using the singularity container with the MEGARes database as a reference and default parameters (--threshold 80 --min 1 --max 100 --samples 5 --skip 5).

### qPCR primer selection and design.

ARG targets were chosen based on initial metagenomic results to capture a range of ARG concentrations. The ARG primer sets were selected based on their use for measuring ARGs in environmental samples (25, 47–49). The primer sets were verified for specificity using NCBI Primer-BLAST and archaea, virus, viroid, and eukaryote databases. Details of the qPCR assays, including primer sequences and annealing temperatures are provided in Table S1 in the supplemental material. qPCRs were carried out on an Eppendorf MasterCyler ep realplex (2) using Fast EvaGreen fast master mix (Biotium, Fremont, CA). The 20-μl reactions were performed following the manufacturer's recommendations, with 0.4 μM forward and reverse primers, 0.625 mg/ml of Ultrapure bovine serum albumin (BSA) (Invitrogen), and 2 μl of diluted DNA extracts. Plates were centrifuged for 2 min at 500 rpm at 4°C before thermocycling. Unpooled sample DNA extracts, with total DNA concentrations ranging from 20 to 50 ng/μl, were diluted 10-fold and 100-fold to detect inhibition from the sample matrix. Inhibition was not observed, and the gene concentrations from both dilutions were averaged. Two technical replicates were performed per diluted sample, and two no-template controls were included on each plate. No template controls were always negative. After amplification, melt curves were performed to confirm the specificity of the reactions. The template for the standard curve consisted of Gblock Fragments (IDT, Skokie, Illinois) with the inserted target sequences taken from a sequence from CARD or NCBI if the primers did not hit the CARD reference gene (Table S1). The qPCR assay limit of detection and limit of quantification were evaluated for each of the ARGs (Table S1) following the European Network of GMO Laboratory Guidelines (50).

### Data availability.

Raw sequence data were deposited to the NCBI BioProject database under BioProject identifier (ID) PRJNA675135 (https://www.ncbi.nlm.nih.gov/bioproject/675135).
