# Erratum for Crossette et al., “Metagenomic Quantification of Genes with Internal Standards”

**DOI:** 10.1128/mBio.01174-21

**Published:** 2021-06-01

**Authors:** Emily Crossette, Jordan Gumm, Kathryn Langenfeld, Lutgarde Raskin, Melissa Duhaime, Krista Wigginton

**Affiliations:** a Department of Civil and Environmental Engineering, University of Michigan, Ann Arbor, Michigan, USA; b Ecology and Evolutionary Biology, University of Michigan, Ann Arbor, Michigan, USA

## ERRATUM

Volume 12, no. 1, e03173-20, 2021, https://doi.org/10.1128/mBio.03173-20. It has come to our attention that text in a paragraph of our introduction misrepresented a referenced study. Below is the original and revised introduction paragraph.

## ORIGINAL TEXT

Incorporating internal standard spike-ins, as commonly used in analytical chemistry, can establish a ratio of metagenomic read abundance to gene copy concentration. Internal standard protocols were first applied to sequencing methods in transcriptomic experiments (RNA-seq) to quantify gene expression, identify protocol-dependent biases, and compare method sensitivity and reproducibility (17). Since then, protocols have been developed for 16S rRNA gene-amplicon (18) metagenome (19), and metatranscriptome (16) sequencing. Previous quantitative metagenomic spike-in studies have performed metagenome assemblies and then mapped short metagenomic reads to the assembled contigs (20). Such assembly-dependent methods are time-intensive and can fail to assemble genomes that harbor ARGs, particularly those of viruses (21) or plasmids and within genomic islands (22, 23), thus increasing false-negative detection rates. Additionally, assemblies can introduce bias toward highly abundant organisms, which are more likely to be assembled correctly (24).

## MODIFIED TEXT

Incorporating internal standard spike-ins, as commonly used in analytical chemistry, can establish a ratio of metagenomic read abundance to gene copy concentration. Internal standard protocols were first applied to sequencing methods in transcriptomic experiments (RNA-seq) to quantify gene expression, identify protocol-dependent biases, and compare method sensitivity and reproducibility (17). Internal standard protocols have since been developed for quantitative 16S rRNA gene-amplicon (18), metagenome (19, 20), and metatranscriptome (16, 20) sequencing. In an early, if not the first, application of internal standards to quantify gene abundances in metagenomic data, Satinsky et al. added known bacterial genomic DNA and mRNA constructs to samples to quantitatively evaluate transcript/gene copy ratios and contrast taxon distributions in free-living and particle-associated microhabitats in the Amazon River Plume (20). Since this early application, the development of a number of high-speed read mapping tools and the drop in sequencing costs provide an opportunity to expand this approach to map orders of magnitude more reads to substantially larger gene databases. With sufficient evaluation of sequencing and read-mapping biases, this approach holds promise to replace low-throughput qPCR measurements with a quantitative metagenomic approach that can simultaneously quantify an unlimited number of gene targets in environmental samples.
