# Supplementary figures and images for: Metagenomic Quantification of Genes with Internal Standards
Source: mBio. 2021 Feb 2;12(1):e03173-20. doi: 10.1128/mBio.03173-20 (PMC7858063; doi:10.1128/mBio.03173-20)

**
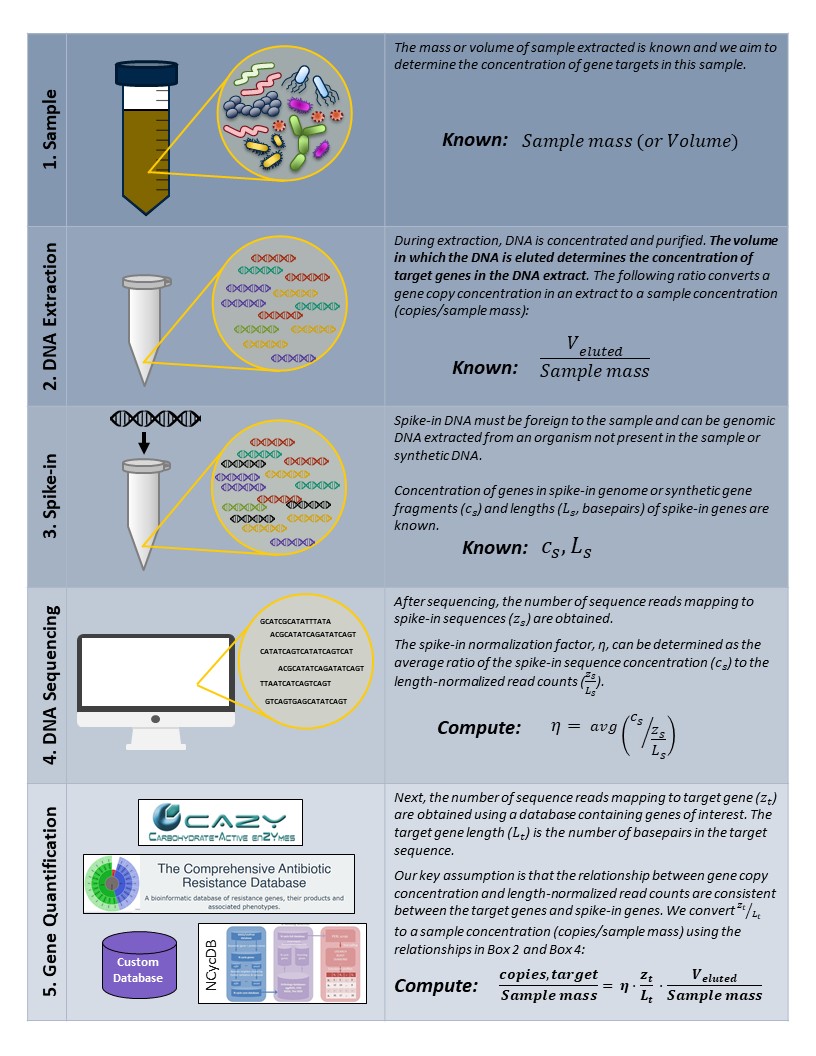
FIG. S1:** Spike-in experimental and bioinformatic approach

Supplement: FIG S1 [file mBio.03173-20-sf001.docx]
