# Supplementary material for: Metagenomic Quantification of Genes with Internal Standards
Source: mBio. 2021 Feb 2;12(1):e03173-20. doi: 10.1128/mBio.03173-20 (PMC7858063; doi:10.1128/mBio.03173-20)
Supplement: FIG S2 [file mBio.03173-20-sf002.docx]

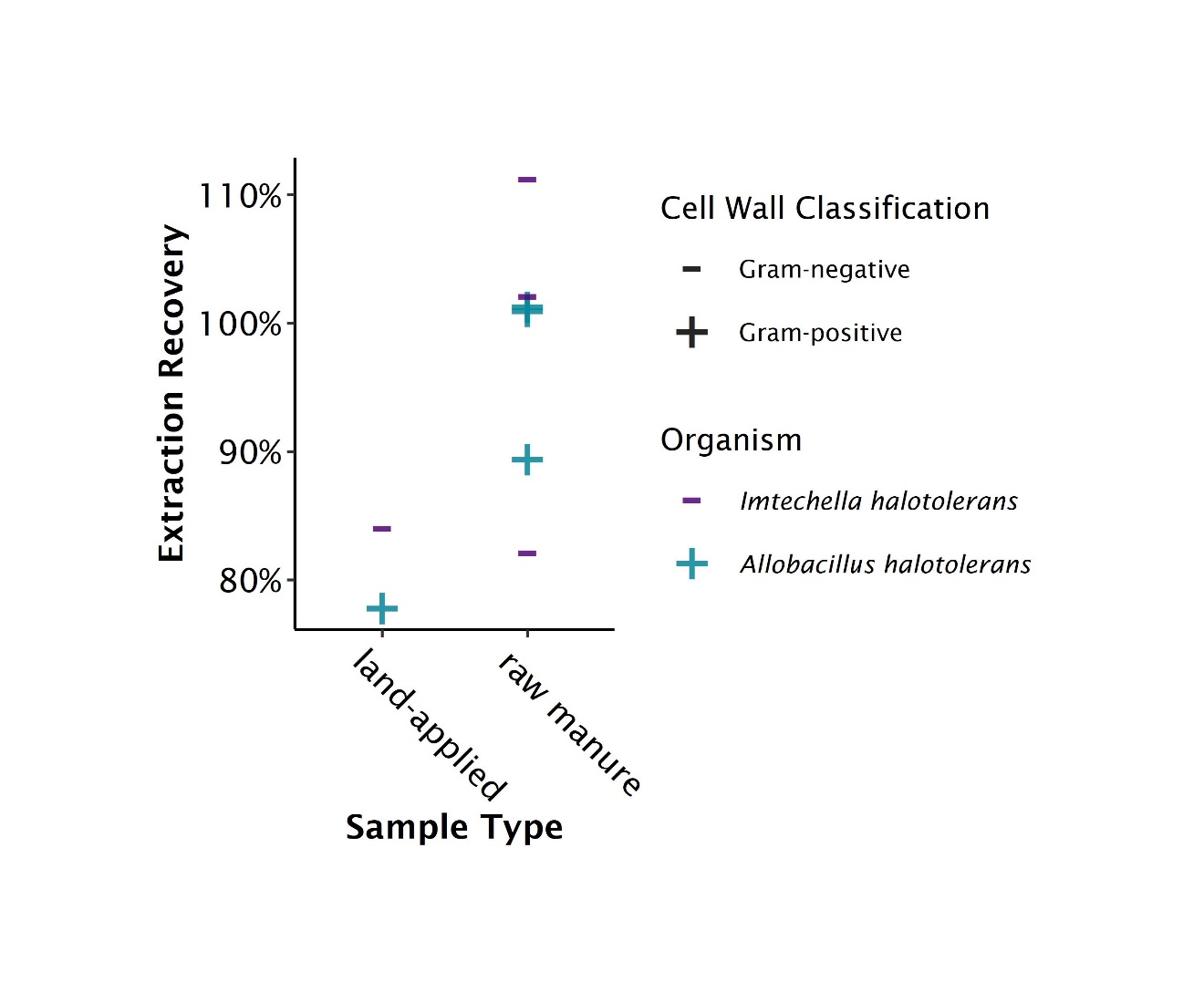


**FIG. S2:** Extraction recovery observed for raw manure and land-applied manure slurry. **TEXT S1.** Describes details of the approach to evaluate extraction recovery.
