# Supplementary material for: Metagenomic Quantification of Genes with Internal Standards
Source: mBio. 2021 Feb 2;12(1):e03173-20. doi: 10.1128/mBio.03173-20 (PMC7858063; doi:10.1128/mBio.03173-20)
Supplement: TEXT S1 [file mBio.03173-20-s0001.docx]

**SUPPLEMENTAL TEXT 1: Extraction Recovery Analysis**

**Spike-in and Extraction**

We validated that our extraction methods provided adequate recovery of DNA and minimal extraction bias between gram-positive and gram-negative bacteria in various dairy manure sample types. 10 µL of ZymoBIOMICS Spike-in Control I (ZYMO Research, Irvine, California), which contains a gram-negative bacterium, *Imtechella halotolerans* (LMG 26483), and a gram-positive bacterium, *Allobacillus halotolerans* (LMG 24826), was spiked into 50-250 mg of land-applied manure slurry and manure stockpile samples. DNA was extracted from the Zymo-containing spikes with the QiaAMP PowerFecal kit (QIAGEN, Germantown, MD).

**Extraction efficiency – qPCR Protocol**

Custom qPCR primers were designed to target a sequence unique to each organism (Table S1) and percent recovery was calculated as the (Expected Gene Copies – Recovered Gene copies) / Expected Gene Copies * 100. qPCR reactions were carried as described in the main manuscript with the following differences. Extracts were diluted 10-fold and 100-fold prior to qPCR to identify potential inhibition. G-Block fragments (IDT, Skokie, Illinois) containing amplicon sequences (reference in Table S1) were used for the qPCR standard curve. Assuming one sequence per CFU or Zymo-reported cell numbers, the expected recovery was the number of organisms spiked into the sample. Recoveries of the *Imtechella halotolerans* and *Allobacillus halotolerans* from the land applied and raw manure samples ranged from 75-110% and did not differ significantly between the gram-positive and gram-negative organisms spiked into the samples (p-value = 0.27, **Fig. S2**). In both sets of extraction experiments, the spiked organisms were not detected in unspiked samples.
