# Supplementary material for: Metagenomic Quantification of Genes with Internal Standards
Source: mBio. 2021 Feb 2;12(1):e03173-20. doi: 10.1128/mBio.03173-20 (PMC7858063; doi:10.1128/mBio.03173-20)
Supplement: FIG S3 [file mBio.03173-20-sf003.docx]

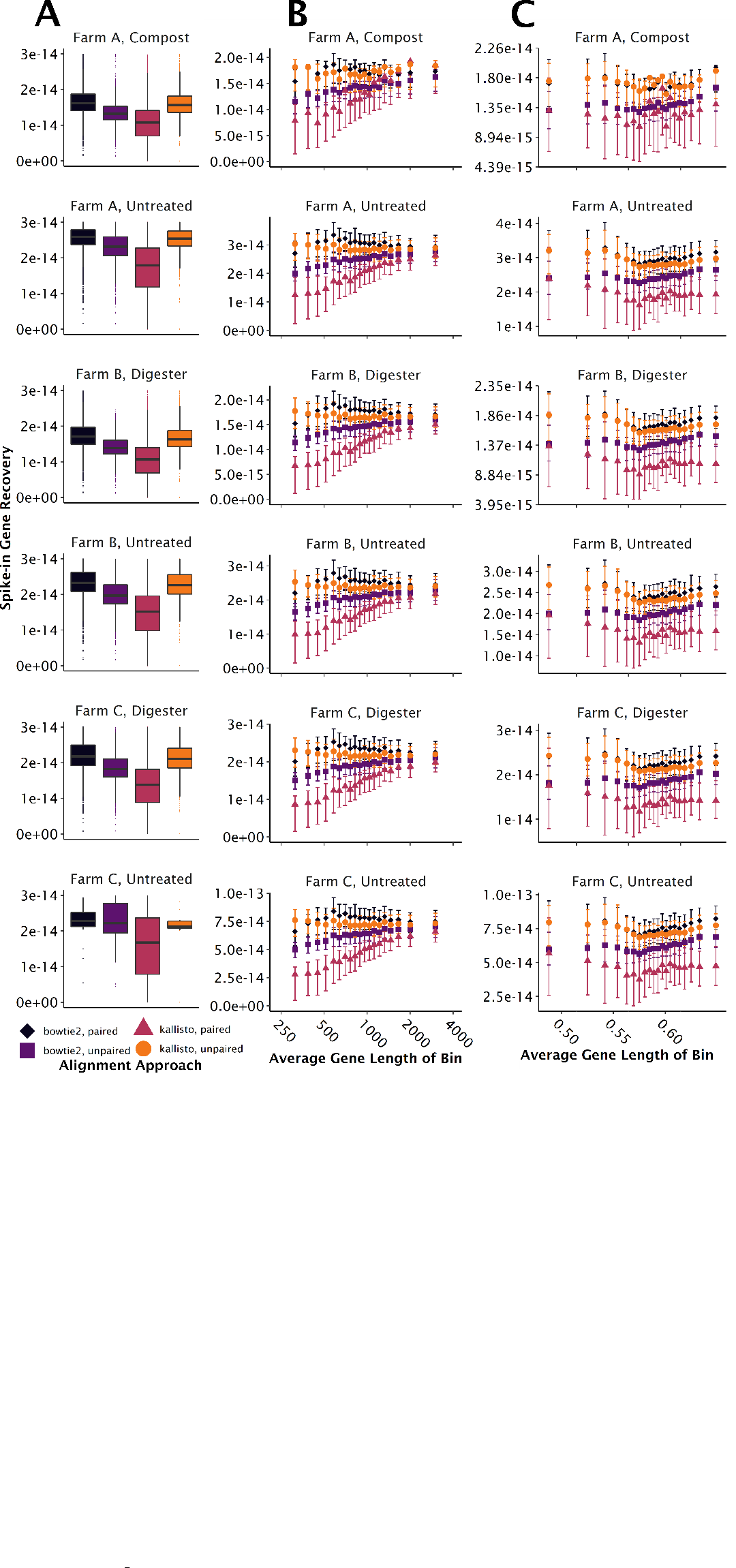


**FIG. S3: A.** Variation in spike-in gene recoveries ($\frac{z_{s,i}}{z_{tot}L_{s,i}}/c_{s,i})$using four mapping approaches. Units of the y-axis ar $1/\frac{gene copies}{\mu L*basepairs}$ where the gene copies/µL are the known copies per DNA extract volume and basepairs are based on the length of the gene. **B and C.** Spike-in gene recovery of *M. hydrocarbonoclasticus* genes from the metagenome across (B) gene lengths and (C) %G+C-contents. Genes are binned into 20 quantiles and extended lines represent the interquartile range for each bin.
