# Supplementary material for: Metagenomic Quantification of Genes with Internal Standards
Source: mBio. 2021 Feb 2;12(1):e03173-20. doi: 10.1128/mBio.03173-20 (PMC7858063; doi:10.1128/mBio.03173-20)
Supplement: FIG S4 [file mBio.03173-20-sf004.docx]

**
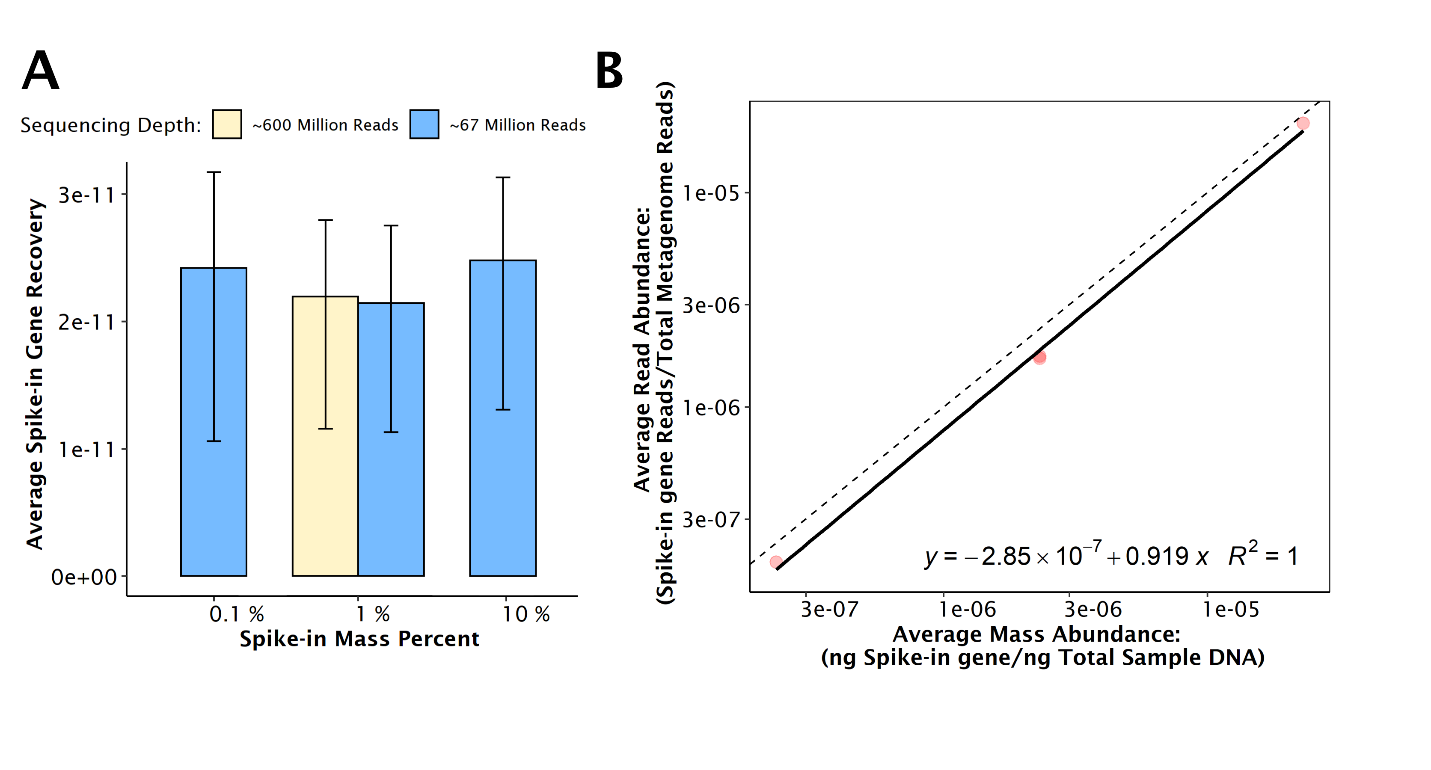
**

**FIG. S4**: **A.** Spike-in gene recovery of internal standards across different concentrations of spike-in. **B.** Correlation of relative number of reads mapped to spike-in genes (z_i_/z_tot_) to spike-in gene mass abundance (mass of Spike-in DNA/Total DNA) for the four same spike-ins in Fig 2A. **Dotted line:** Theoretical 1:1 relationship, **Solid line** linear regression. Analysis performed using the Farm C Digester Sample.
