# Supplementary material for: Metagenomic Quantification of Genes with Internal Standards
Source: mBio. 2021 Feb 2;12(1):e03173-20. doi: 10.1128/mBio.03173-20 (PMC7858063; doi:10.1128/mBio.03173-20)
Supplement: TEXT S2 [file mBio.03173-20-s0002.docx]

## SUPPLEMENTAL TEXT 2: Read-mapping validation

## Spike-in gene recoveries are dependent on read mapping method, gene size, and %G+C content.

We examined the impact of %G+C content and gene length on the recovery of the *M. hydrocarbonoclasticus* genes spiked into DNA extracted from dairy manure samples and sequenced using an Illumina HiSeq4000. This quantitative metagenomic approach uses a known ratio of read abundance to gene concentration to convert read abundances of target sequences to target gene concentrations. Therefore, it is critical to assess factors that can impact the read recovery rate such as gene length and %G+C content. Recovery was defined as the ratio of observed read abundances to the known input concentration of each spike-in gene. Specifically, the ratio consisted of the length-normalized number of reads that mapped to each of the *M.* *hydrocarbonoclasticus* spike-in genes divided by the total number of metagenomic reads in the sample ($\frac{z_{s,i}}{z_{tot}L_{s,i}}$) over the known concentration of that gene in the sequenced extract ($c_{s,i},$gene copies/µL of DNA extract). Herein, this recovery ratio ($\frac{z_{s,i}}{z_{tot}L_{s,i}}/c_{s,i})$ is referred to as the “spike-in gene recovery” and reflects recoveries through sequencing and read annotation. A uniform spike-in gene recovery across gene %G+C content and gene length would demonstrate that the sequencing and mapping approach is not biased by these factors.

We mapped reads to the *M. hydrocarbonoclasticus* genes using two mapping tools, kallisto and Bowtie2. Kallisto and Bowtie2 were used since they are common tools for read alignment in metagenomic samples^1–3^ and employ two different algorithms for read assignment. Kallisto is a pseudo-alignment tool that uses k-mer hashing to find exact matches between reads and references, and Bowtie2 is a Burrows-Wheeler transform-based alignment. The stringency of read-mapping parameters was relaxed by mapping reads as individual reads (unpaired) to observe the impact of paired and unpaired read-mapping on recovery of the spike-in genes. The four mapping approaches resulted in significantly different average gene recoveries for all samples (Fig. S3 A, ANOVA, F<2x10^-16^ for all samples). Kallisto with paired mapping resulted in the lowest recoveries and is likely too strict of a read assignment approach.

Gene length and %G+C-dependent biases in gene recovery within each read mapping approach were next identified. Recoveries decreased as gene lengths decreased for all the approaches except for kallisto in the unpaired setting (Fig. S3 B). The spread of recoveries, measured as the inter-quartile ranges of recoveries across gene bins, decreased with increasing gene size (Fig. S3 B). The recoveries leveled-out at gene sizes above 600 basepairs with the Bowtie2-paired mapping approach, but never leveled out for the kallisto-paired and Bowtie2-unpaired mapping approaches. As gene lengths approach the library insert size (here, 450 basepairs), read pairs are less likely to map since a critical fraction of the read pair may extend beyond the target gene reference sequence. Depending on the mapping approach used, this limits read mapping to the spike-in reference gene sequence and thus biases quantification.

Patterns of spike-in gene recovery across %G+C contents were consistent for all mapping approaches (Fig. S3 C). Average spike-in gene recoveries decreased as average %G+C content increased. A minimum average spike-in gene recovery was reached around 55% G+C content and then increased again as %G+C contents increased. Large biases caused by %G+C were not expected in this study, since previous studies using Illumina platforms found PCR-free library preparation minimized this bias.^4,5^ Minimum and maximum spike-in gene recoveries in the %G+C content bins within mapping tools differed at most by 10% with kallisto in the paired setting in all samples. For the other three tools, the average percent difference in minimum and maximum gene recovery across bins was between 4-5%.

It is important to note that in complex environmental samples, some genes in the *M. hydrocarbonoclasticus* genome may share sequence similarity with genes of microorganisms present in the samples, whereby false-positives could artificially inflate read mapping rates. To assess if outliers with high read mapping drive the recovery ratio, we compared the mean and median spike-in gene recoveries. The mean and median values differed by less than 8% and, therefore, it is unlikely that false-positives have a major impact on the recovery. However, to completely avoid incorrect read mapping to the spike-in genome, synthetic, non-coding DNA can be used as an internal standard spike-in.^4^ The kallisto-unpaired read mapping approach was selected for the sequencing efficiency calculation because it consistently demonstrated the lowest spike-in gene recovery bias for the spike-in genes across gene lengths.

**Spike-in gene read abundances correlated with spike-in gene mass abundances over three orders of magnitude**

To examine the impact of the spike-in gene concentration on measured read abundances (z_i_/z_tot_) and to ensure the spike-in quantitative approach was valid across a range of gene concentrations, we compared the spike-in gene recoveries at spike-in concentrations that spanned three orders of magnitude. Specifically, one sample extract was separately spiked with the *M. hydrocarbonoclasticus* such that the gene concentrations were 8x10^4^, 8x10^5^, and 8x10^6^ copies per µL (which equates to 0.1%, 1%, and 10% of total sample DNA mass as the spike-in genome based on fluorometric quantification (Qubit, Thermo Scientific)). Gene recoveries were reproducible when run at the same spike-in abundance but sequenced at different depths (Fig. S4A). Spike-in gene recoveries were consistent across the different mass spikes (Fig. 2A) and read abundances of internal standard genes were proportional to the gene concentration of spike-in internal standard DNA (linear regression R^2^ = 1; Fig. S4B).

In addition to confirming that read abundances increase proportionally with the known gene concentrations, these results indicate the general range of gene detection limits. At our sequencing effort in this experiment (50 million reads per sample), no reads mapped to 95 of the 4272 genes in the sample with the lowest spike-in gene concentrations (8x10^4^ copies of each gene per µL of DNA extract). Therefore, we can estimate the detection limit of detection range of 10^4^ gene copies/µL extract, or about 3.2x10^4^ gene copies/mg sample. Despite all genes being present at the same gene copy concentration, the mass concentrations in the extract ranged from 9x10^-7^ to 6x10^-5^ ng DNA/μL, with a mean of 8x10^-6^ ng DNA/μL. The concentrations of the 95 undetected *M*. *hydrocarbonoclasticus* genes were on the lower end of these ranges, from 9x10^-7^ to 1x10^-5^ ng DNA/μL, with a mean of 3x10^-6^ ng DNA/μL. For comparison, our qPCR limits of detection were as low as 5 gene copies/µL DNA extract and our limits of quantification ranged from 10-60 gene copies/µL DNA extract, depending on the assay (Table S1). Based on this assessment, qPCR remains the more appropriate method for quantifying genes at low abundances. However, as the cost of sequencing declines, the ability to capture more sequences from a sample will facilitate lower detection limits. To achieve detection limits approaching those of qPCR in this study, we would have needed a 1000-fold higher sequencing depth (equivalent to 50 billion reads).

(1) Nayfach, S.; Pollard, K. S. Toward Accurate and Quantitative Comparative Metagenomics. *Cell* **2016**, *166* (5), 1103–1116. https://doi.org/10.1016/j.cell.2016.08.007.

(2) McCall, C.; Xagoraraki, I. Comparative Study of Sequence Aligners for Detecting Antibiotic Resistance in Bacterial Metagenomes. *Lett. Appl. Microbiol.* **2018**, *66* (3), 162–168.

(3) Schaeffer, L.; Pimentel, H.; Bray, N.; Melsted, P.; Pachter, L. Pseudoalignment for Metagenomic Read Assignment. *Bioinformatics* **2017**, *33* (14), 2082–2088.

(4) Hardwick, S. A.; Chen, W. Y.; Wong, T.; Kanakamedala, B. S.; Deveson, I. W.; Ongley, S. E.; Santini, N. S.; Marcellin, E.; Smith, M. A.; Nielsen, L. K.; et al. Synthetic Microbe Communities Provide Internal Reference Standards for Metagenome Sequencing and Analysis. *Nat. Commun.* **2018**, *9* (1), 1–10. https://doi.org/10.1038/s41467-018-05555-0.

(5) Browne, P. D.; Nielsen, T. K.; Kot, W.; Aggerholm, A.; Gilbert, M. T. P.; Puetz, L.; Rasmussen, M.; Zervas, A.; Hansen, L. H. GC Bias Affects Genomic and Metagenomic Reconstructions, Underrepresenting GC-Poor Organisms. *Gigascience* **2020**, *9* (2), giaa008.

(6) Alcock, B. P.; Raphenya, A. R.; Lau, T. T. Y.; Tsang, K. K.; Bouchard, M.; Edalatmand, A.; Huynh, W.; Nguyen, A.-L. V; Cheng, A. A.; Liu, S.; et al. CARD 2020: Antibiotic Resistome Surveillance with the Comprehensive Antibiotic Resistance Database. *Nucleic Acids Res.* **2019**, *48* (October 2019), 517–525. https://doi.org/10.1093/nar/gkz935.
