# Supplementary material for: Metagenomic Quantification of Genes with Internal Standards
Source: mBio. 2021 Feb 2;12(1):e03173-20. doi: 10.1128/mBio.03173-20 (PMC7858063; doi:10.1128/mBio.03173-20)
Supplement: FIG S5 [file mBio.03173-20-sf005.docx]

**
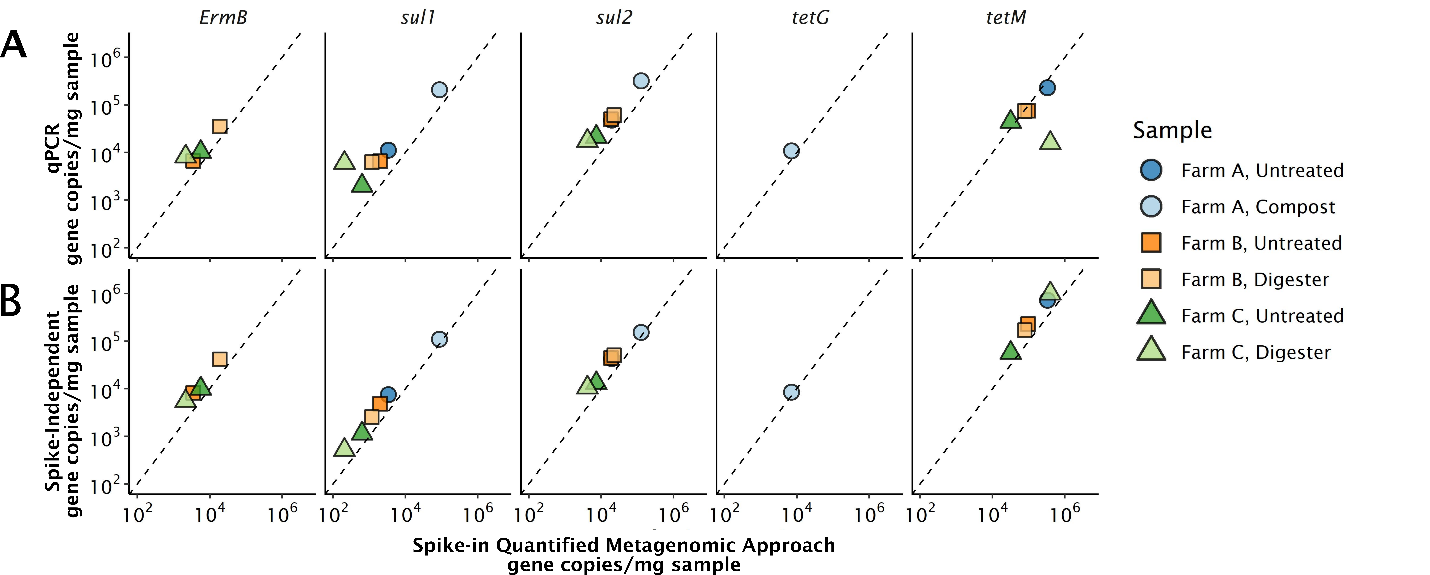
**

**FIG. S5:** Comparisons of the gene quantification approaches using AMR++ tool for assigning reads to ARG reference sequences. The dotted line is a 1:1 line that represents theoretical perfect correlation between approaches. **A.** Spike-in-quantified metagenomic absolute abundance approach vs. qPCR; **B.** Spike-in-quantified metagenomic absolute abundance approach vs. spike-independent, 16S rRNA gene-based metagenomic approach. *ErmB* was not detected in the Farm A, Samples with AMR++ but was detected in the Farm A Compost sample with qPCR. *TetG* was detected with qPCR in all samples but not the quantitative metagenome approach.
