# Supplementary material for: Metagenomic Quantification of Genes with Internal Standards
Source: mBio. 2021 Feb 2;12(1):e03173-20. doi: 10.1128/mBio.03173-20 (PMC7858063; doi:10.1128/mBio.03173-20)
Supplement: TABLE S1 [file mBio.03173-20-st001.docx]

**TABLE S1:** qPCR primers used in this study with annealing temperatures and results of LOD, LOQ, and efficiency assessments.

| **Target** | **Primer Set (5`-3`)** | **Amplicon** | **Ref** | **LOD** | **LOQ** | **LOD**^a^ | **AT^b^** | **ɳ^c^** | **r^2^** |
| --- | --- | --- | --- | --- | --- | --- | --- | --- | --- |
|  |  | **Accession #** |  | copies/µL | | copies/mg |  |  |  |
| *sul1* | CGCACCGGAAACATCGCTGCAC  GAAGTTCCGCCGCAAGGCTCG | gb\|JF969163\|+\|1054-1893\|sul | 1 | 20 | 60 | 8 | 62 | 98% | 0.997 |
| *sul2* | TCCGGTGGAGGCCGGTATCTGG  CGGGAATGCCATCTGCCTTGAG | NG_048113.1 | 1 | 10 | 20 | 4 | 58 | 88% | 0.999 |
| *TetG* | GCAGAGCAGGTCGCTGG  CCYGCAAGAGAAGCCAGAAG | gb\|AF133139\|+\|1-1176\|tet(G) | 2 | 5 | 20 | 2 | 53 | 92% | 0.999 |
| *TetM* | CCGTTGGGAAGTGGAATGC  TCCGAAAATCTGCTGGGGTA | NC_004116.1:c929374-927455 | 3 | 5 | 10 | 2 | 59 | 88% | 0.999 |
| *ErmB* | AAAACTTACCCGCCATACCA  TTTGGCGTGTTTCATTGCTT | gb\|AF242872\|+\|2132-2878\|ErmB | 4 | 5 | 20 | 4 | 50 | 91% | 0.999 |
| *16S rRNA* | ACTCCTACGGGAGGCAG  ATTACCGCGGCTGCTGG | CP026677.1:1353823-1354031 | 5 | - | - | - | 54 | 88% | 1.000 |
| *Imtechella halotolerans* | TTACCCGCCGACAGATTAGC  TTGCGTCCTATGGGGCTTTCT | LMG 26483 | TS^d^ | - | - | - | 60 | 82% | 0.997 |
| *Allobacillus halotolerans* | TCGCTCCAAACCAGTCCATC  ACACCAGGGTAAGTGACTGC | LMG 24826 | TS | - | - | - | 60 | 79% | 0.998 |

*a. LOD in sample mass = gene copies/µL * 100 µL DNA extract / Sample mass (0.250 g), b. Annealing Temperature, c. Efficiency from standard curve, d. TS = this study*

**Primer References:**

1. Pei, R.; Kim, S.-C.; Carlson, K. H.; Pruden, A. Effect of River Landscape on the Sediment Concentrations of Antibiotics and Corresponding Antibiotic Resistance Genes (ARG). *Water Res*. 2006, 40 (12), 2427–2435.

2. Aminov, R.; Chee-Sanford, J.; Garrigues, N.; Teferedegne, B.; Krapac, I.; White, B.; Mackie, R. I. Development, Validation, and Application of PCR Primers for Detection of Tetracycline Efflux Genes of Gram-Negative Bacteria. *Appl. Environ. Microbiol*. 2002, 68 (4), 1786–1793. https://doi.org/10.1128/AEM.68.4.1786.

3. Luo, Y.; Mao, D.; Rysz, M.; Zhou, Q.; Zhang, H.; Xu, L.; Alvarez, P. J. J. Trends in Antibiotic Resistance Genes Occurrence in the Haihe River, China. *Environ. Sci. Technol*. 2010, 44 (19), 7220–7225. https://doi.org/10.1021/es100233w.

4. Knapp, C. W.; Dolfing, J.; Ehlert, P. A. I.; Graham, D. W. Evidence of Increasing Antibiotic Resistance Gene Abundances in Archived Soils since 1940. *Environ. Sci. Technol*. 2010, 44 (2), 580–587. https://doi.org/10.1021/es901221x.

5. Fierer, N.; Jackson, J. A.; Vilgalys, R.; Jackson, R. B. Assessment of Soil Microbial Community Structure by Use of Taxon-Specific Quantitative PCR Assays. *Appl. Environ. Microbiol*. 2005, 71 (7), 4117–4120. https://doi.org/10.1128/AEM.71.7.4117-4120.2005.
