# Supplementary material for: Metagenomic Quantification of Genes with Internal Standards
Source: mBio. 2021 Feb 2;12(1):e03173-20. doi: 10.1128/mBio.03173-20 (PMC7858063; doi:10.1128/mBio.03173-20)
Supplement: TABLE S2 [file mBio.03173-20-st002.docx]

**TABLE S2:** Allele frequencies of single nucleotide variants (SNV) compared to the *tetM* forward primer sequence aligned using Bowtie2. The base in the forward primer and position is provided as the column headers. Depth refers to the average read abundance.

|  | Primer Position, Base (5’-3’) | | | | | | |
| --- | --- | --- | --- | --- | --- | --- | --- |
| Sample | 3, G | 6, G | 9, A | 12, T | 16, A | 18, G | Depth |
| Farm A, Untreated | 79%, A* | 79% A | 77% G | 77% C | 77% C | 76% A | ~2600 |
| Farm A, Compost | 71% A | 71% A | 29% G | 71% C | 71% G | 71% A | 7 |
| Farm B, Untreated | 79% A | 78% A | 21% G | 78% C | 78% G | 77% A | ~1150 |
| Farm B, Digester | 73% A | 73% A | 27% G | 72% C | 71% G | 71% A | ~450 |
| Farm C, Untreated | 63% A | 62% A | 38% G | 60% C | 60% G | 56% A | ~650 |
| Farm C, Digester | 99% A | 99% A | 01% G | 99% C | 99% G | 99% A | ~4000 |

*79% of reads that mapped to the former primer position 3 contained an “A” rather than a “G”
